# Foix-Chavany-Marie syndrome secondary to bilateral traumatic operculum injury

**DOI:** 10.1007/s00701-018-3702-x

**Published:** 2018-10-17

**Authors:** Richard Digby, Adam Wells, David Menon, Adel Helmy

**Affiliations:** Division of Neurosurgery, Addenbrooke’s Hospital, University of Cambridge, Cambridge, CB2 0QQ England

**Keywords:** Foix-Chavany-Marie, Pseudobulbar palsy, Frontal aslant tract, Supplementary motor area syndrome

## Abstract

This report describes a case of a 62-year-old man who developed Foix-Chavany-Marie syndrome subsequent to traumatic brain injury. The initial presentation of the syndrome was profound loss of voluntary control of orofacial muscles, causing a loss of speech and impairment of swallow. Over subsequent months, a remarkable recovery of these functions was observed. The natural history of FCMS in this case was favourable, with good improvement in function over months. Furthermore, the pattern of bilateral opercular injury was more readily recognised on MRI than on CT, supporting the role of MRI in cases of traumatic brain injury.

**Electronic supplementary material:**

The online version of this article (10.1007/s00701-018-3702-x) contains supplementary material, which is available to authorized users.

## Introduction

Foix-Chavany-Marie syndrome (FCMS) is a form of pseudobulbar palsy named for the French authors who first reported it in 1926 [3]. Typically, it is described as a loss of voluntary control of palatal, glossal, pharyngeal, masticatory and oral muscles, but with preservation of reflex movements. As such, production of speech, chewing and swallowing are impaired. It most commonly occurs secondary to bilateral opercular stroke [8, 12], but other cases reported in the literature include following insular glioma resection [2, 6] and unilateral opercular contusions following traumatic brain injury [7]. In some cases, the syndrome is transient, resolving over the course of days [6]; in others, little improvement is seen over longer time frames [5, 8].

## Case history

**Fig. 1:**
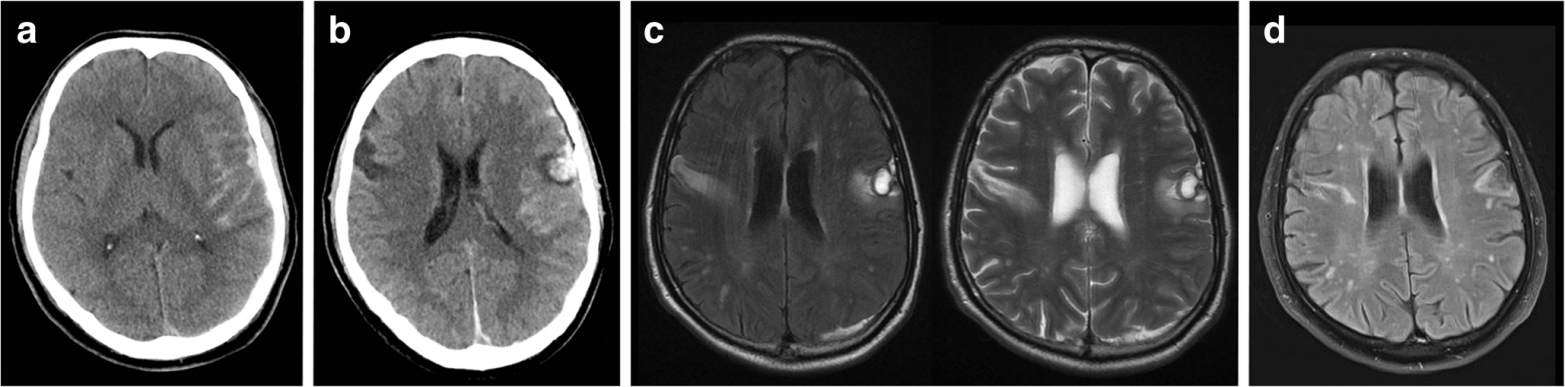
Neuroradiological imaging. CT scan on the day of the injury (**a**) and 2 days later (**b**). MRI scan performed at 2 weeks demonstrated bilateral opercular lesions (**c**). Progress MRI at 13 months demonstrated persistent FLAIR signal change in these regions (**d**)

A 62-year-old right hand-dominant retired male academic with a history of hairy cell leukaemia in remission suffered a traumatic brain injury after falling from his bicycle. His Glasgow Coma Scale (GCS) score was 3/15 at the scene and improved to 11/15 (E2V2M6) by the time of his arrival to hospital. He was intubated to facilitate his computed tomography (CT) head scan, which demonstrated subarachnoid haemorrhage within the left Sylvian fissure and extending over the left cerebral convexity (Fig. 1a). He was subsequently transferred to a tertiary institution with neurocritical care support for ongoing management.

The following day, he was obeying commands and extubated; however, he was dysphasic. By day 2 after injury, he was drowsier and developed respiratory failure and was subsequently re-intubated. A repeat CT scan demonstrated interval development of a haemorrhagic contusion within the left opercular region and a new corresponding right inferolateral frontal hypodensity (Fig. 1b). A right frontal intracranial pressure (ICP) was inserted, and his neurological and respiratory condition slowly improved to the point that he was extubated a second and final time on day 13 post-injury.

In the days following the second extubation, the patient still could not communicate verbally. It was noted that he had impaired upper airways reflexes and could not protrude his tongue. Further examination revealed oropharyngeal paraesthesia, an absent gag reflex, tongue paralysis and bilaterally abducted vocal folds. Other findings included bilateral lower face, tongue and pharyngeal paralysis with limited movement in his forehead, but with preservation of his jaw jerk. Magnetic resonance imaging (MRI) brain scan at this point excluded a brainstem pathology and confirmed bilateral traumatic opercular lesions (Fig. 1c), and the radiological findings coupled with the neurological deficit suggested FCMS secondary to traumatic brain injury (TBI).

Management consisted of nasogastric feeding and chest physiotherapy. Despite a spontaneous cough, he had persistent absence of voluntary function of his lower cranial nerves. Initial serial fibre-optic endoscopic examination of swallow (FEES) assessments demonstrated gradual improvement of laryngeal function with resolution of vocal fold paralysis with persistent motor and sensory pharyngeal dysphagia. By 5 weeks post-injury, his tongue movements were sufficient to lick lips, and facial movements had improved to the point that he was able to mouth words and be understood. At 6 weeks, he was could phonate enough that verbal communication was possible; however, swallowing was still impaired. It was only at this point that a mild cognitive impairment was recognised, and he was subsequently transferred to a rehabilitation facility. He was seen twice as an outpatient clinic; on the first review, 6 months post-injury, it was noted that his speech had improved significantly and that his swallow had improved enough to tolerate a soft diet. His cognition had improved to the point that he was able to resume co-authoring peer reviewed papers; however, he was still troubled by fatigue and short-term memory deficits. By 12 months, his speech had returned to almost normal, however was slower than prior to the injury and required more concentration. Nevertheless, the strategies provided via a speech therapy programme had allowed the patient to return to public-speaking engagements and lecturing; audio samples of which are provided in online supplementary material [supplementary audio files 1, 2, 3]. He was tolerating a normal diet, with little or no evidence of persisting FCMS.

## Discussion

This case is an example of remarkable recovery from Foix-Chavany-Marie syndrome. While a relatively rare syndrome, it is most commonly seen following bilateral opercular stroke [8, 12]. This particular case was precipitated by a specific pattern of TBI, namely bilateral damage to the frontal opercula. Initial CT scanning (Fig. 1a) only indicated a left opercular contusion; however, MR imaging clearly demonstrated a FLAIR signal abnormality on the contralateral side. In this case, the region affected by the bilateral lesions would be consistent with damage to the frontal aslant tract, a structure that connects the supplementary motor area with Broca’s area [1]. Damage to this tract has been implicated as potentially responsible for the clinical picture of FCMS, as it is thought to contribute to control of orofacial movements [6].

This case demonstrated clinical FCMS subsequent to traumatic injury of the anterior opercula bilaterally. Anatomically, this is consistent with classical descriptions of the pattern of injury understood to give rise the syndrome [3, 8]. The need for bilateral injury is thought to be due to the redundancy in the initiation mechanisms that underlie the syndrome. However, this particular case showed some lower cranial nerve signs in the early phase that are not part of FCMS as described in the strictest sense, such as the absent gag reflex and bilaterally abducted vocal cords. This may be due to the traumatic origin of the injury in this case; the nature of traumatic brain injury means that these are rarely isolated injuries. As FCMS is most commonly described as arising following ischaemic stroke, it is therefore likely that FCMS subsequent to traumatic brain injury will have scope for variations in the specific clinical signs manifested, depending on the exact pattern of injury in the particular case.

FCMS has many parallels with the much more commonly encountered supplementary motor area syndrome (SMAS). SMAS is characterised by a loss of volitional movement contralateral to the site of injury (usually surgical) in the posterior medial frontal lobe immediately anterior to the primary motor cortex. It is easily recognised clinically by the profound contralateral plegia with maintenance of reflex movements [9]. The supplementary motor area has a somatotopic organisation, has reciprocal connections with the striatum, is activated in advance of primary motor cortex in volitional movement and is therefore considered part of the initiation pathway for internally directed actions [4]. Critically, there are also connections, via the frontal aslant tract [11], to the frontal operculum and we hypothesise that FCMS is a specific corollary of SMAS that affects the face, pharynx, larynx and face.

Recognition of FCMS in this case resulted from integrating clinical details of the neurological deficit with radiological findings, particularly from the MRI scan. Although the MRI was performed over 2 weeks post-injury and subsequent to attempts at extubation, it may be the case that early recognition of this pattern of injury could be helpful in raising clinical suspicion of FCMS, enabling anticipation of the challenges to respiratory and nutritional management that FCMS presents. While the treatment for FCMS itself is primarily supportive for the bulbar symptoms, and therefore early recognition of the syndrome would be unlikely to change the management of the condition itself, it would facilitate more accurate counselling of the patient and family in light of the favourable prognosis. CT remains the first-line imaging modality in TBI; however, MRI is increasingly becoming a useful adjunct, being more sensitive for certain parenchymal lesions or diffuse axonal injury [10]. While the development of the right inferolateral frontal hypodensity is visible on CT in Fig. 1b, the MRI images in Fig. 1c are superior for demonstrating the nature of the lesions in this case. As such, this example provides support for the role of MRI scanning in TBI cases, especially when clinical details are not fully explained by CT findings. As the role of MRI scanning in TBI develops secondary to technological advances and reducing costs, it may be the case that FCMS is recognised more frequently than it is at present.

In this particular case, the patient showed progressive improvement in condition over subsequent months; in the first few weeks, he regained the ability to mouth words, but later, his cognition improved such that he could co-author academic papers. Over the same period, his speech also improved to the extent that he could resume public speaking engagements. Overall, this can be interpreted as a favourable natural history in the long term. Previous reports of FCMS and of many different aetiologies, have reported variable outcomes; these range from early recovery of function over a matter of days [6] to poor recovery over long periods [5]. This case shows a significant recovery of function, albeit over a timespan of months following the initial trauma. It is possible that the FCMS secondary to traumatic brain injury carries a more favourable prognosis, compared to infarction, as the underlying brain has more scope for recovery. Furthermore, analogous to the SMAS, the recovery is aided by the greater scope for plasticity in the supplementary motor area than in primary motor cortex.

## Electronic supplementary material

ESM 1 (MP3 1922 kb)

ESM 2 (MP3 1972 kb)

ESM 3 (MP3 2448 kb)
